# Structure of Stacked Aggregates of Semiflexible Rings Under Spherical Confinement: A Computational Study

**DOI:** 10.3390/polym18050602

**Published:** 2026-02-28

**Authors:** Xiaolin Zhou, Yifan Qin, Youfei Xie, Andrey G. Cherstvy

**Affiliations:** 1 College of Physics and Optoelectronic Engineering, Harbin Engineering University, Harbin 150001, China; xlzhou@hrbeu.edu.cn (X.Z.);; 2 Key Laboratory of In-Fiber Integrated Optics, Ministry of Education, Harbin Engineering University, Harbin 150001, China; 3 Key Laboratory of Photonic Materials and Device Physics for Oceanic Applications, Ministry of Industry and Information Technology of China, Harbin Engineering University, Harbin 150001, China; 4 Department of Physics, School of Science, Westlake University, Hangzhou 310024, China; xieyoufei@westlake.edu.cn; 5 Institute of Physics and Astronomy, University of Potsdam, 14476 Potsdam-Golm, Germany

**Keywords:** spherical confinement, semiflexible ring polymers, molecular dynamics, stacked aggregates

## Abstract

How ordered and mutually independent are semiflexible ring polymers (RPs) confined to a spherical cavity of variable radius? By varying the cavity radius, we systematically investigate the effect of the confinement size on the conformations of RPs using the coarse-grained molecular dynamics simulations. The results reveal that as the bending energy increases, the RPs exhibit a transition from a purely flexible coil to an elongated oblate-shaped object and, eventually, to a disk-like conformation. Simultaneously, the stacked aggregates composed of adjacent, mutually nearly parallel, semiflexible RPs emerge for stiffer chains. We find that the structural modulation of the stacked aggregates is regulated by the confinement size. For the conditions of strong confinement ($R<2R_{g}$, where $R_{g}$ is the radius of gyration of an RP), the semiflexible RPs undergo peculiar deformations and twisting that lead to disruption of the stacked aggregates. At $R\approx 2R_{g}$, the average number of the RPs per stack reaches a maximum. Concurrently, the order of spatial alignment of all semiflexible RPs is maximized with the global orientational-order parameter reaching the value S≈0.79. As the cavity radius further increases, at $R>3R_{g}$, the semiflexible RPs gain greater mobility resulting in diverse orientations of the aggregates being formed, with the order parameter dropping to S≈0.05. These findings provide important quantitative insights for future applications of the RPs, i.e., in micro- and nanodevice assembly.

## 1. Introduction

Ring polymers (RPs)—as a unique class of macromolecular systems characterized by their end-free topology—exhibit fundamental differences from their linear counterparts in terms of, i.e., conformational evolution [1,2,3,4], aggregation behavior [5,6,7], and macroscopic physical properties [8,9,10,11]. Recently, the RPs as well as knotted chains [12] have become a core research direction in the field of topological polymeric studies in macromolecular physics. The breakthrough development of precision-synthesis techniques—exemplified by the applications of atom-transfer radical polymerization [13,14,15,16], ring-opening metathesis polymerization [17,18,19], and topology-selective cyclization reactions [20,21,22]—has enabled the researchers to achieve a controllable preparation of the cyclic polymers with tailored lengths and rigidities. This laid a solid experimental foundation for systematic studies of the aggregation behavior of various RPs.

Semiflexible RPs—serving as a key intermediate system between flexible and rigid RPs—possess moderate bending energies. Their stacking behavior is governed by the interplay of multiple factors (i.e., the chain rigidity [23,24,25,26,27,28], topological constraints [23,24], intermolecular van der Waals forces [29,30], π–π interactions [31,32,33,34], local heterogeneities in the composition, and external environmental conditions [35,36]). This separates them from the entropy-driven random entanglement and the aggregation-limiting characteristic of flexible RPs [23,37,38,39], while also distinguishing them from the energy-driven and highly ordered close-packing features of the rigid RP motifs. Consequently, semiflexible RPs form aggregated or stacked structures that exhibit both topological specificity and structural tunability [26,40], thereby establishing themselves as a unique and irreplaceable model for elucidating the structure–property relationships between the topology, aggregated structure, and macroscopic performance of the RPs.

The stacking behavior of semiflexible RPs is prevalent across various cutting-edge fields, including the biomolecular assembly [31,32], fabrication of advanced polymer composites [33], processing of organic optoelectronic devices [41], and supramolecular self-assembly [34]. The orderliness, stability, spatial configuration, and dynamic evolution of various stacked RP structures directly determine the core physical properties of the respective materials (such as their mechanical, optical, and charge-transport characteristics, as well as their rheological characteristics).

In biological systems, stacking and condensation of natural semiflexible ring biomacromolecules—such as cyclic DNAs and ring-shaped proteins—are the key processes for genetic-material packaging [42,43] and enzymatic function [44]. Tightly wound DNA toroids can also be mentioned here [45,46]. The stacking modes of semiflexible RPs are regulated by both the chain rigidity [23,24,26,28] and by specific intermolecular interactions [47,48]. Elucidating these mechanisms provides a theoretical basis for certain aspects of gene therapy and biomimetic-material design.

In synthetic materials, the stacking mode of semiflexible conjugated polymers during thin-film formation directly influences the degree of π-π stacking within the film, which, in turn, governs the charge mobility and device efficiency in organic field-effect transistors [49] and in organic solar cells [50]. In the design of topological polymer composites, the co-stacking behavior of semiflexible RPs with the linear chains and with nanoparticles can modulate the interfacial compatibility and phase structure of the composites, thereby enabling a targeted optimization of their mechanical strength and thermal stability [51,52].

Furthermore, in a multitude of confined environments—such as nanopores [53,54], planar interfaces [55,56,57], and microcapsules [58,59,60]—the stacking behavior of semiflexible RPs can be dynamically modulated by external geometric constraints and by interfacial interactions. This leads to the formation of stacked aggregates with distinct spatial architectures, offering novel pathways for fabricating polymeric materials with well-defined ordered structures on the micro- or nanoscale.

In recent years, advancements in molecular simulation techniques—such as molecular dynamics, Monte Carlo, dissipative particle dynamics, and coarse-grained simulation methods—combined with the application of advanced characterization tools—including atomic force microscopy, transmission electron microscopy, small-angle X-ray scattering, and neutron scattering—have enabled the researchers to elucidate the stacking mechanisms of semiflexible RPs both at molecular and mesoscopic scales. These tools enable investigation of how different factors—such as chain rigidity, areal number density, confining interfaces, and blend composition—regulate the emerging stacking structures.

Extensive computer simulations have been performed on the systems consisting of multiple semiflexible RPs [61,62]. The results have demonstrated that the chains spontaneously form characteristic *stacked aggregates*. With the periodic boundary conditions and at comparatively high polymer concentrations, the semiflexible RPs can adopt regular conformations with columnar tubular architectures. This aggregation arises from a synergistic effect of the excluded volume and orientational correlations in such densely packed environments, which collectively drive the polymer chains to adopt a quasi-parallel alignment to minimize their mutual steric repulsion.

Furthermore, a number of researchers have found that in bulk melts, semiflexible RPs can form caged structures, and the topology-specific stacking can induce a non-classical glass transition—termed a topological glass state—which significantly slows down the relaxation dynamics in the system [27,29,40,63]. In confined environments, strongly repulsive flat walls force semiflexible RPs to stack into oblate shapes *parallel* to the surface. In contrast, attractive walls—due to the competition between the chain–wall interactions and topological constraints—can lead to partially *perpendicular* chain alignment. Moreover, wall-induced confinement significantly restricts the formation of large-scale stacked aggregates [64]. In blends of semiflexible RPs with linear chains, threading events and entropic interactions between the two components induce the formation of highly ordered co-stacking domains. The rigidity of the RPs modulates the strength of threading, thereby influencing the degree of order in such co-stacked structures [51].

Staño *et al.* used the molecular dynamics simulations and found out that at nanoscale the columnar stack formation by PRs is a common system characteristic, in which the counterion valence is a key factor in modulating the stack stability. Specifically, high concentrations of several monovalent counterions (e.g., Na^+^ and K^+^) induce intra-stack charge separation and a high free-energy penalty; the osmotic pressure of the free ions outweighs the entropy-driven stacking tendency of the RPs, forcing RP deformation to reduce the charge repulsion and, ultimately, stack disintegration.

In contrast, some trivalent ions facilitate chain-entropy-driven aggregation via the so-called ion bridging (simultaneous binding to multiple RPs), which further stabilizes stacks and promotes longer columnar stack formation by the RPs [40]. The discussion of these findings has employed the anisotropic effective interaction model [23,40,62] to quantitatively describe the stacking behavior of semiflexible RPs and to characterize the phase-transition processes of the stacked structures. These findings not only enriched the aggregation theory of semiflexible RPs, but also laid a solid foundation for their practical applications.

Despite significant advances in studying stacked semiflexible RPs, the formation and evolution of stacked aggregates under confinement are still unclear, as the cavity size critically influences chain conformations. A prime example is the packaging of DNA and RNA genomes within viral capsids [65,66,67]. For *highly flexible* single-stranded nucleic acids, confinement often results in a “disordered”, loose packing due to the electrostatic attraction to the oppositely charged inner surface of viral capsids [65,68,69], with the chains staying still largely Gaussian in their conformational statistics. In contrast, *stiff* double-stranded DNAs inside the bacteriophages form highly organized patterns [46,70,71]. Inside a phage, a tightly packed DNA arranges into locally parallel near-hexagonal structures, often leading to a global toroidal order imposed by the protein capsid [45,72,73,74,75,76,77]. Notably, the internal radius of many phage capsids—e.g., ≈28 nm for T7 λ-phage, ≈27 nm for T7 P22 capsid, ≈42 nm for T4 phage [78,79,80]—is smaller than ∼50 nm of the B-DNA persistence length, necessitating extreme external bending and twisting deformations of the DNA.

In this paper, we use coarse-grained molecular dynamics simulations combined with theoretical analysis to systematically investigate the effects of confinement size and chain rigidity on the stacking structures and conformational evolution of the RPs. Furthermore, we establish a quantitative theoretical framework suitable to describe the stacking behavior of semiflexible RPs, which enables us to characterize these stacked structures. Our work advances the aggregation theory of semiflexible RPs and provides theoretical guidance for the structural design and targeted fabrication of functional materials based on semiflexible RPs.

The plan of the paper is as follows. In Section 2, we start with the description of the theoretical model and of the simulation algorithms. In Section 3, we present the main results and provide a detailed discussion of the role of the model parameters. In Section 4, the conclusions of the current investigation are presented.

## 2. Model and Methods

The physical systems investigated below consist of RPs confined inside rigid spherical cavities of a varying radius *R*. The three-dimensional Cartesian (after Descartes) coordinates {x,y,z} of the beads forming a hard-sphere container satisfy the geometric definition of a sphere,

(1) $$x^{2}+y^{2}+z^{2}=R^{2}.$$

**Figure 1:**
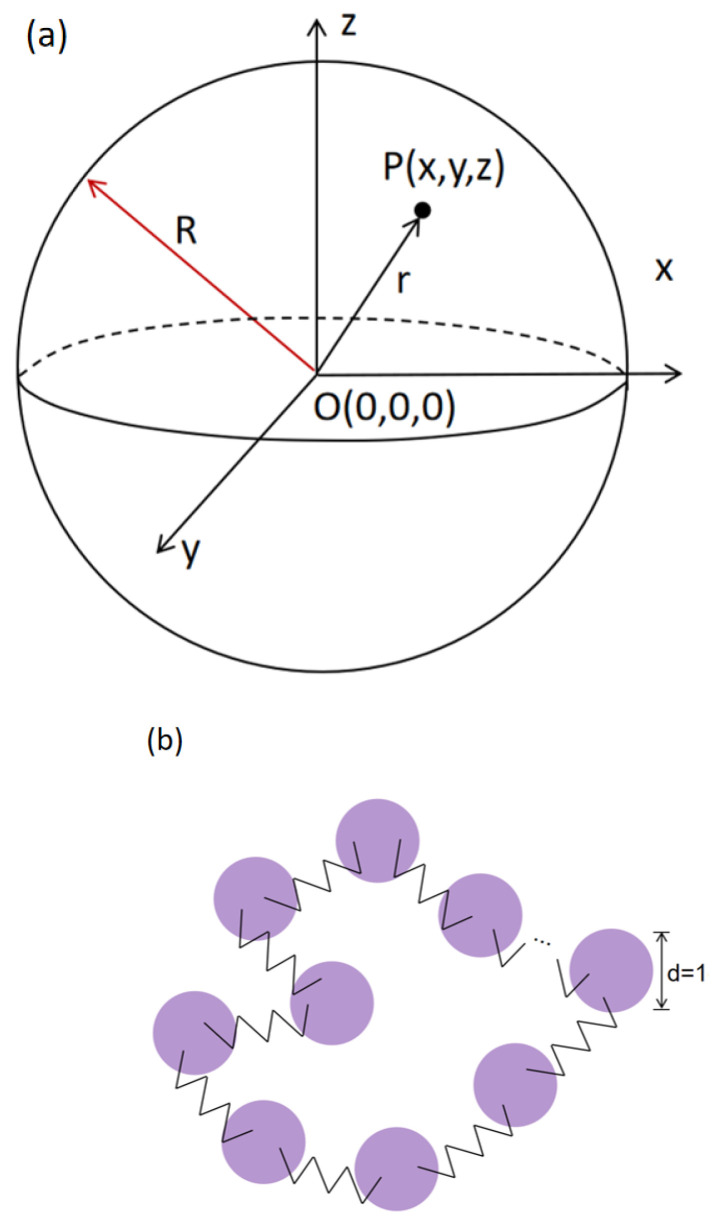
Schematic illustration of (**a**) a spherical container of radius *R* and of (**b**) an RP modeled via the bead-spring model.

The container possesses a uniform smooth surface, with its center located at the origin {x=0,y=0,z=0}, see Figure 1a, ensuring that all subsequent observations and analyses of the molecular spatial positions are based on the same reference. To investigate the effects of different spatial scales on the system properties, the radius *R* is set to multiple values, specifically in the range R=4÷20σ.

The intra- and inter-molecular interactions of each RP are described via a potential energy composed of three terms,

(2) $$U=U_{\mathrm{FENE}}+U_{\Theta }+U_{\mathrm{LJ}}.$$

Specifically, (i) the bond potential $U_{\mathrm{FENE}}$ in Equation (2) characterizes the energy variation due to stretching of covalent bonds between the adjacent monomers; (ii) the angular potential $U_{\Theta }$ accounts for the energy contribution from the bending deformations of the chain backbone; and, lastly; and (iii) the nonbonded potential $U_{\mathrm{LJ}}$ captures the weak interactions between the unbonded monomers. The potential energy (2) comprehensively encompasses the dominant energetic mechanisms governing each RP under spatial confinement.

In detail, the Finite-Extensible Nonlinear-Elastic (FENE) bond potential given by [81,82]

(3) $$U_{\mathrm{FENE}}\left(r\right)=-\frac{1}{2}kR_{0}^{2}\log\left[1-{\left(\frac{r}{R_{0}}\right)}^{2}\right]$$

describes the tensile elasticity of the covalent bonds between the neighboring beads, with a spring constant used in the simulations $k=30k_{B}T/\sigma^{2}$ and the equilibrium bond distance $R_{0}=1.5\sigma$. These parameter values in this conventional model are widely adopted in the community for simulating the RPs.

The rigidity of an RP is characterized by a bond-angle potential expressed mathematically as

(4) $$U_{\Theta }\left(\Theta_{ijk}\right)=K_{b}\left[1-\cos\left(\theta -\Theta_{ijk}\right)\right].$$

Here, θ denotes the equilibrium bond angle between the adjacent segments. For the RPs with the chain length L=30σ, the geometric constraint of the ring closure requires the equilibrium bond angle to satisfy the natural condition [28,30,83,84] $\theta =180^{\circ }$. The variable $\Theta_{ijk}$ refers to the actual angle formed by three consecutive beads {i,j,k} (specifically, the angle between the ji bond and the jk bond). The bending constant $K_{b}$ in Equation (4)—measured hereafter in units $k_{B}T$—modulates the chain rigidity: larger $K_{b}$ values increase the energy cost of angular deformations, enhancing the bending resistance. This parameter is widely applied in studying the conformational evolution and dynamics of semiflexible polymers [47,48,50,51,52,53]. In this work, the values of $K_{b}$ range from 0 to 50, corresponding to fully flexible and semiflexible chains, respectively.

Non-bonded interactions are described by a widely used truncated Lennard–Jones (LJ) 6–12 potential [54,55]

(5) $$U_{\mathrm{LJ}}\left(r\right)=\left\{\begin{array}{cc} 4\epsilon \left[{\left(\frac{\sigma }{r}\right)}^{12}-{\left(\frac{\sigma }{r}\right)}^{6}+\frac{1}{4}\right], & r\leq r_{c},\\ 0, & r>r_{c}. \end{array}\right.$$

To simplify the system and to avoid any interference from the attractive interactions between the chain and the container wall as well as from long-range interactions, we here use the standard cutoff radius $r_{c}=2^{1/6}\sigma$. This ensures that both the inter-RP and RP–container interactions are purely repulsive.

Below, the RP is formed by connecting the ends of a linear chain into a stable closed-loop structure; see a schematic illustration in Figure 1b. To ensure the reproducibility of the model, each monomer of an RP is defined as a spherical bead of diameter d=1. The adjacent monomers are coupled via the elastic connections (3) and (4) with fixed elastic coefficients. These interactions not only maintain the structural integrity of the chain, but also accurately reproduce its elastic deformations under diverse external influences, thereby providing a reliable framework for subsequent investigations of conformational changes of the RPs.

At the initial stage of the simulations, the RPs are placed inside a rigid spherical container in a random uniform manner, ensuring that no overlap between the RPs or of RPs with the container wall exists. In this study, we fix the monomer-number density at $\rho \approx 0.6\sigma^{-2}$; it is calculated as

(6) ρ=LN/V,

where *V* is the volume of the spherical container, *L* is the number of monomers per chain, and *N* is the total number of the RPs in the system. Thus, for different container radii *R*, the corresponding *N* for each system is recalculated via Equation (6). Specific parameters of the simulations are summarized in Table 1, while all the parameters are given in Table S1 of the Supporting Information (SI).

To quantify the structure of confined RPs, we systematically calculate the mean radius of gyration $\langle R_{g}\rangle$ under various conditions of spatial confinement. As a fundamental physical quantity characterizing the spatial extension of a polymer, it is defined as [85,86,87]

(7) $$R_{g}=\sqrt{\langle R_{g}^{2}\rangle }=\sqrt{\frac{1}{N}\sum_{i=1}^{N}\frac{1}{L}\sum_{j=1}^{L}{\left(r_{ij}-r_{\mathrm{CM},i}\right)}^{2}},$$

where $r_{\mathrm{CM},i}$ denotes the coordinates of the center of mass (CM) of the *i*th RP, $r_{ij}$ represents the coordinates of the *j*th monomer on the *i*th RP. As follows from Equation (7), the displacements of the chain monomers from the CM are averaged along each RP and then among all *N* RPs in the system.

For a polymer chain of *L* monomers, its gyration tensor $T_{\alpha \beta }$—which is a 3×3 symmetric matrix—is defined as

(8) $$T_{\alpha \beta }=\frac{1}{L}\sum_{i=1}^{L}\left(r_{i,\alpha }-r_{\alpha ,\mathrm{CM}}\right)\left(r_{i,\beta }-r_{\beta ,\mathrm{CM}}\right),$$

where α and β are {x,y,z}. By diagonalizing this tensor, a set of three eigenvalues $\left\{\lambda_{1},\lambda_{2},\lambda_{3}\right\}$ is obtained, typically ordered as $\lambda_{1}\geq \lambda_{2}\geq \lambda_{3}$.

Our simulation scheme adopts a two-step ensemble protocol: the initial brief relaxation in the *NVE* microcanonical ensemble to rapidly drive the system out of its initial nonequilibrium state, which is followed by simulations in the *NVT* canonical ensemble for a prolonged sampling at a constant temperature *T*. Specifically, the *NVT* phase involves $10^{8}$ molecular dynamics steps, with the first $5\times 10^{7}$ steps dedicated to reaching the equilibrium. The subsequent $5\times 10^{7}$ steps are then used for data production. The trajectory data are sampled every $10^{4}$ steps, and each parameter set is simulated with 10 independent replicates to ensure statistical reliability. All the results are then averaged over the sampled frames and trials.

All physical quantities reported below are given in the reduced units, namely $k_{B}T=1$, σ=1, and m=1 for the energy, length, and mass, respectively. The integration time step is $\tau =0.001\tau_{0}$, where $\tau_{0}=\sigma \sqrt{m/\varepsilon }$ is the characteristic time scale. All the simulations are performed using the open-source LAMMPS (Large-Scale Atomic/Molecular Massively Parallel Simulator) software [88]. These approaches and simulation methods are similar to those recently used by (some of the) authors for the analysis of ordering of separated fragments of semiflexible chains under confinement [36,51,89]. We also refer the reader here to the *theoretical* investigations of polymers under confinement and of chain bending inside the cavities [69,90,91,92].

## 3. Results and Discussion

### 3.1. Conformational Geometry of RPs

We confine a system of RPs with a high monomer number density ρ within a rigid spherical cavity, as depicted in Figure 1a. Serving as a rigid boundary, the spherical container of radius *R* undergoes neither deformations nor displacements. To comprehensively investigate the chain conformations and the spatial arrangements under different degrees of confinement, the radius *R* is systematically varied, from the values smaller than the radius of gyration of an RP to the values significantly larger than $R_{g}$, specifically in the range R=4÷20.

**Figure 2:**
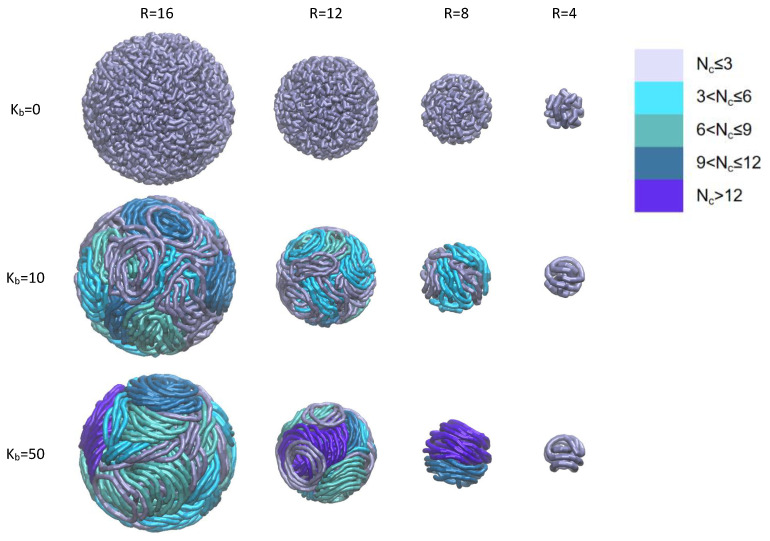
Typical snapshots of the RPs with varying bending rigidities $K_{b}$ confined in spherical cavities of varying radii *R*. The columns from left to right correspond to R=16,12,8,4, respectively. The rows represent the polymer rigidities: $K_{b}=0$ (purely flexible), $K_{b}=10$ (weakly semiflexible), and $K_{b}=50$ (semiflexible chain). The following color-coding scheme is adopted to classify the number of chains in each aggregate: dispersed rings with 1÷3 RPs are labeled in ice blue, small stacks with 4÷6 RPs are labeled in celeste, medium stacks with 7÷9 RPs are labeled cyan, large stacks with 10÷12 are labeled in blue, and, finally, very large stacks with >12 RPs are labeled in the violet color. The chosen number of categories is not critical here, serving solely the illustrative purposes.

In Figure 2, we show some snapshots of a typical simulation of statistically representative individual configurations of the RPs, visualized for the polymers of different rigidities and for confining spheres of varying radii. From left to right in Figure 2, the radius of the spherical cavity decreases gradually from R=16 to R=4. From top to bottom in Figure 2, the bending rigidity of the RPs increases from $K_{b}=0$ to $K_{b}=50$. Additional snapshots from the simulations reflecting a broader range of these model parameters are provided in Figure S1 of the SI.

We find that in weakly confined environments (see, e.g., the first column of Figure 2 at R=16), as the ring rigidity increases from $K_{b}=0$ to $K_{b}=50$, the spatial conformations of the chains exhibit notable and systematic changes. Flexible RPs with $K_{b}=0$ adopt nearly randomly coiled conformations due to their thermal fluctuations and intramolecular interactions. With increasing $K_{b}$, the resistance of RPs to deformations grows, causing the RPs to gradually turn into a more expanded state. For high-rigidity conditions, at $K_{b}=50$, the RPs eventually form very regular, disc-like circular-type structures. Such structures are reminiscent of snail-like aggregates formed upon complex formation of the double-stranded DNAs with the electrostatically attractive cationic lipid membranes [93].

Concurrently, an increasing bending rigidity promotes interchain aggregation leading to the formation of the so-called “stacked aggregates”, the structures composed of multiple mutually aligned RPs. To better visualize these aggregates and to classify the number of RPs in each stack, in Figure 2 a rich color-coding scheme is adopted. This scheme illustrates the differences in the aggregate-size distribution under given conditions.

As the cavity radius is reduced to R=8, see the third column of Figure 2, for the RPs with the stiffness $K_{b}=50$, the average number of chains within the stacked aggregates reaches a maximum. This phenomenon arises from the synergy between the degree of confinement and the interchain interactions: namely, moderately decreasing the cavity radius from R=16 to R=8 reduces the available space for a free movement of the RPs, thereby increasing the probability of collisions and of contacts of the neighboring chain segments. Moreover, it does not significantly disrupt the disc-like conformation of such rigid RPs: the chains maintain a regular morphology necessary for parallel stacking or alignment. Consequently, this fact promotes the formation of large-scale aggregates of stacked RPs. The specific distribution of the number of RPs per aggregate, the average size of the aggregates and their statistical significance under these conditions are quantitatively analyzed in Section 3.2 below through numerical computations, such as those of the radial distribution functions, the statistics of the chain counts within the aggregates, and the measures of the orientational order of the RPs.

When the degree of confinement is further increased, reaching R=4, as shown in the fourth column of Figure 2, in this strongly confined environment the chain conformations and the aggregate structures of the RPs undergo significant changes. For the cases of $K_{b}=10$ and $K_{b}=50$, the spatial compression exerted by the container gives rise to disruption of the well-defined disc-like structures of the RPs, which start to exhibit severe deformations, local bending, and segmental distortions. The stacked aggregates completely disintegrate and the system of RPs starts to be dominated by individual deformed chains. This demonstrates a *disruptive effect* of the excessive confinement on the formation of ordered aggregates of RPs.

**Figure 3:**
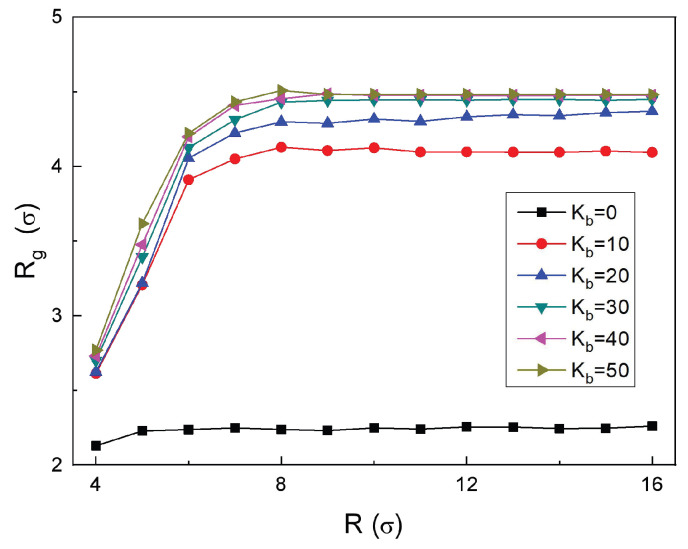
Dependence of the radius of gyration $\langle R_{g}\rangle$ of the RPs on the spherical-cavity radius *R* (both in units of σ) for varying polymer rigidities.

For the case $K_{b}=0$, the RPs are fully flexible, allowing free folding in space. The variations in the radius *R* of the confining sphere have no significant influence on the values of $\langle R_{g}\rangle$, which remain nearly constant at $\langle R_{g}\rangle \approx 2.24$, as shown by the black data points in Figure 3. This is because the conformations of flexible RPs are entropy-driven and these chains can autonomously adjust their conformations to adapt to the confined space they reside in. Thus, their overall extension is independent of the degree of external confinement, a characteristic directly reflected by the $\langle R_{g}\rangle$ values.

This behavior is further verified by the subsequent calculation of the key physical quantities characterizing the anisotropic extension of polymers in 3D: for short flexible chains of length L=30 used here, the maximal eigenvalue derived via the diagonalization of the radius-of-gyration tensor (8) ranges from $\lambda_{1}\approx 2.54$ to $\lambda_{1}\approx 3.13$ (consistently under various confining conditions). These values are smaller than the radii of all spherical cavities in our simulation system. This further confirms from a microscopic perspective that the overall extension of flexible RPs is minimally affected by the external confinement. All relevant results for flexible RPs are summarized in Table S2 in the SI.

For $K_{b}=10$, the chain rigidity increases, restricting its ability to fold freely and promoting more extended spatial conformations. However, this rigidity-driven conformational evolution is significantly modulated by spatial confinement. Under a strong confinement, e.g., at R<8, the physical boundaries of the container exert a pronounced compression, preventing the RPs from fully expanding into the conformations preferred from the viewpoint of minimal bending energy alone. As a result, the nominal volume occupied by the confined chains is reduced and the $\langle R_{g}\rangle$ remains relatively small: e.g., $\langle R_{g}\rangle \approx 2.61$ for R=4, as follows from Figure 3.

As the cavity radius increases to R=8, the compressive effects weaken and the rigidity-driven chain expansion becomes dominant, leading to an increase of $\langle R_{g}\rangle$ to $\langle R_{g}\rangle \approx 4.12$. For the situation R>8, the confining sphere becomes sufficiently large to accommodate *largely unperturbed* conformations of such semiflexible chains. As a result, a plateau at $\langle R_{g}\rangle \approx 4.12$ emerges; see the red circles and red curve in Figure 3. These results for moderately rigid RPs clearly demonstrate the synergistic regulation of the chain conformations by polymer rigidity and external confinement.

Upon a further increase in polymer rigidity, up to $K_{b}=50$, the RPs adopt stable, disc-like, nearly circular shapes. For comparison, the theoretical value for $R_{g}$ for an ideally circular ring is $R_{g}=L/\left(2\pi \right)\approx 4.77$. As the sphere radius increases, the available space gradually becomes sufficient to stabilize some disc-like conformations. Ultimately, the $\langle R_{g}\rangle$ of the RPs stabilizes at $\langle R_{g}\rangle \approx 4.48$; see the brown triangular data points in Figure 3.

The specific eigenvalues of the gyration tensor (8) for semiflexible RPs with $K_{b}=50$ under a spherical confinement of different radii *R* are summarized in Table 2. To maintain the focus here, the data generated in simulations for *other* bending rigidities (in the range from $K_{b}=0$ to $K_{b}=40$) and for additional parameter combinations are presented in Tables S2–S6 of the SI.

To analyze the shapes in detail, we computed the quantifiers of prolateness and asphericity [61,94,95,96,97] of the RPs. The prolateness—serving as a sensitive indicator of the spatial conformation of semiflexible RPs—can be calculated from the eigenvalues $\lambda_{1,2,3}$ as [61,94,95,96,97]

(9) $$p=\frac{\left(2\lambda_{1}-\lambda_{2}-\lambda_{3}\right)\left(2\lambda_{2}-\lambda_{1}-\lambda_{3}\right)\left(2\lambda_{3}-\lambda_{1}-\lambda_{2}\right)}{2{\left(\lambda_{1}^{2}+\lambda_{2}^{2}+\lambda_{3}^{2}-\lambda_{1}\lambda_{2}-\lambda_{1}\lambda_{3}-\lambda_{2}\lambda_{3}\right)}^{\frac{3}{2}}}.$$

The value of p=−1 ($\lambda_{1}=\lambda_{2}>\lambda_{3}$) indicates a perfectly oblate conformation of an RP, while p=1 ($\lambda_{1}>\lambda_{2}=\lambda_{3}$) corresponds to a perfectly prolate shape.

**Figure 4:**
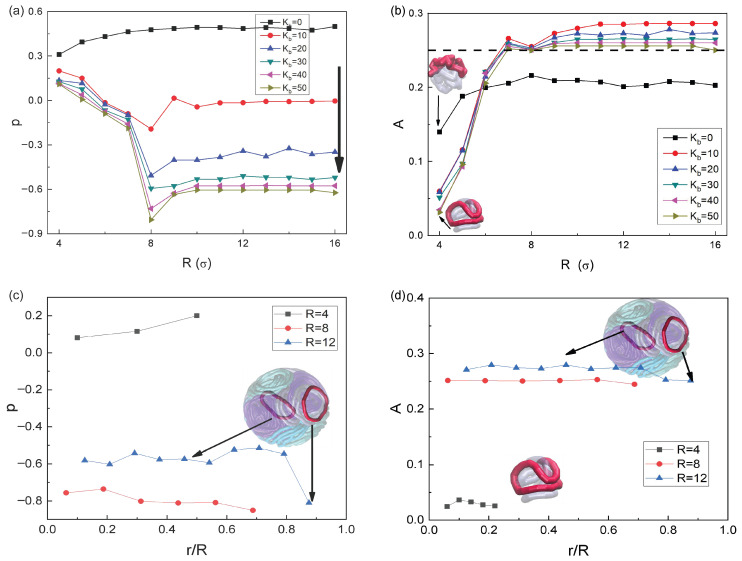
(**a**) Prolateness of the RPs versus the confinement radius *R* of spherical cavities shown for a set of chain-rigidity values $K_{b}$. The direction of the *p* variation with $K_{b}$ in panel (**a**) is indicated by the black arrow. (**b**) Asphericity of the PRs as a function of confinement radius *R* for varying $K_{b}$. (**c**) Spatial distribution of the prolateness parameter of the RPs with $K_{b}=50$ at different locations within the confined sphere. In panel (**c**), the particular values of *p* are associated with the indicated rings both inside the cavity and on the inner surface of the sphere. (**d**) Spatial distribution of the asphericity parameter for the RPs with $K_{b}=50$ at different locations within the sphere.

As Figure 4a shows, with increasing bending energy, the value of *p* progressively approaches −1. This trend reflects a gradual transition of the collapsed flexible RPs to semi-rigid RPs with regular circular morphology, as higher bending energy suppresses chain deformations and promotes circular conformations. Actually, Bernabei *et al.* have found [61] that under unconfined periodic boundary conditions the high-density semiflexible RPs in the bulk adopt oblate conformations with p=−1.

For the systems with $K_{b}=50$ (see the brown triangles in Figure 4a), under strong confinement with the radii of R<8, a pronounced compression effect by the cavity leads to a significant deviation from the p=−1 value. When the confinement radius reaches R=8, the RPs no longer undergo any noticeable deformation stemming from the external restrictive surface typically “sanctioning” the polymers. Meanwhile, the RPs tend to form stacked aggregates aligned in parallel to one another within the cavity. This morphological feature is evident in the simulation snapshots presented in the third column of Figure 2. The rings are fully expanded, adopting a regular oblate conformation with a corresponding prolateness value of p≈−0.80, which is quite close to the limiting value of p=−1.

As the cavity radius further increases R>8, the available free volume inside the cavity rises remarkably, thus leading to the formation of multi-chain aggregates with anisotropic characteristics. Some RPs migrate toward the inner wall of the cavity and adopt surface-adsorbed conformations, which are significantly different from those in the cavity interior. Specifically, in the cavity interior—despite a sufficient overall space—the interchain-crowding effect hinders the RPs from maintaining circular conformations. In contrast, the RPs near the inner cavity wall align along this inherently curved surface; the coupled effect of spatial confinement and of curvature of the cavity drives these near-wall RPs to form more nearly circular conformations.

To compare the conformational differences of the RPs in different regions, we calculate locally their prolateness. For convenience in comparing the characteristics of different regions for various *R*, we normalize the radial distance to the cavity radius (serving as the characteristic scale) and use this ratio as the abscissa axis to investigate the prolateness of the PRs in different cavity regions. The results of these simulations are presented in Figure 4c.

Taking the system with R=12 (the blue curve in Figure 4c) as an example, the results demonstrate that the PRs in the cavity interior—affected by the interchain crowding effect—struggle to maintain a circular conformation (a prolateness value of p≈−0.57, deviating significantly from that of an ideal oblate conformation p=−1), see the inset in Figure 4c. In contrast, the PRs near the inner wall can align along the curved surface of the cavity and form a nearly circular conformation under the effect of spatial confinement and curvature support, with a prolateness of p≈−0.80. These results clearly indicate that as the confinement radius *R* increases further, the proportion of internal rings increases (at the same particle number density), which ultimately leads to larger *p* values.

To further investigate the structural properties of the RPs, we calculate their asphericity [25,94,98,99]

(10) $$A=\frac{{\left(\lambda_{1}-\lambda_{2}\right)}^{2}+{\left(\lambda_{1}-\lambda_{3}\right)}^{2}+{\left(\lambda_{2}-\lambda_{3}\right)}^{2}}{2{\left(\lambda_{1}+\lambda_{2}+\lambda_{3}\right)}^{2}}.$$

It is a quantitative index characterizing the anisotropy degree of three-dimensional conformations and of geometric shapes of a polymer. When A=0 the chain presents an ideal spherical structure, while larger values of *A* indicates a higher degree of anisotropy.

As shown in Figure 4b, in the region for R>5 in the case $K_{b}=0$, the RPs are purely flexible and adopt cloud-like conformations. In this case, the three eigenvalues of the gyration tensor are comparable, as shown in Table S2 in the SI, ultimately leading *A* to attain its minimal value (see the black data points and curve in Figure 4b). For the situation of $K_{b}=10$, the RPs exhibit a gradual expansion. However—under the dual constraints of high monomer-number density and spherical confinement—the RPs tend to form prolate asymmetric conformations (see Table S3 in the SI). This is manifested via a remarkable disparity in the respective eigenvalues yielding $\lambda_{1}>\lambda_{2}\gg \lambda_{3}$ as well as with the *A* values reaching their maximum (the red curve in Figure 4b).

For the case $K_{b}>10$, a further increase of the bending stiffness enables the RPs to form stable and regular oblate conformations. In this situations, the difference between the $\lambda_{1}$ and $\lambda_{2}$ reduces, as shown in Tables S4–S6 in the SI, the structural regularity is improved compared to that at $K_{b}=10$, the anisotropy degree of the RPs decreases and the *A* values show a slight reduction. Notably, regardless of the increase in rigidity of the RPs, rigid chains cannot achieve a relatively uniform spatial packing typical for purely flexible RPs. Even when forming the oblate cyclic conformations, the spatial distribution of the RPs is essentially confined to a quasi-two-dimensional plane, rather than being homogeneously distributed in space, resulting in values of *A* for the rigid RPs consistently higher than for flexible RPs.

At R=8, we get A≈0.25, which is close to the theoretical value of the asphericity of the ideal disk-like semiflexible-RP conformation and also consistent with the aspect-ratio results presented in Figure 4a. Furthermore—after normalizing the radial distance using the sphere radius and plotting this normalized ratio along the *x*-axis—we calculated the average asphericity *A* of the RPs in different cavity regions. The results presented in Figure 4d confirm that at R=12—as shown by the blue curve in Figure 4d—the RPs adjacent to the inner wall of the cavity tend to adopt circular conformations with A≈0.25, consistent with the results in Figure 4c.

Under the strong-confinement condition, at R<5, the asphericity of RPs with different rigidities decreases. For $K_{b}=50$, the conformations of semiflexible RPs are predominantly governed by the spatial constraint due to the cavity, and the RPs adhere to the spherical cavity to form curved tennis-ball-like (see Ref. [90]) conformations (see the lower inset in Figure 4b), leading to a small difference among the eigenvalues and a reduced *A* value.

For $K_{b}=0$, the purely flexible RPs exhibit extremely low local inhomogeneity (Table S2 in the SI). The closed topological structure of the RPs causes partial chain segments to undergo compact local folding and to form microscale aggregation domains, while other chain segments experience slight stretching due to steric compression and thus form microscale stretching domains (the upper inset in Figure 4b). Consequently, the asphericity of this system is higher than that of the system at $K_{b}=50$ at a strong confinement.

### 3.2. Stacked Aggregate Structures of RPs

In Section 3.1, we established that the RPs distribute themselves within a spherical cavity, adopting predominantly the oblate and prolate conformations. In practice, due to a high density of the chain monomers, the confined semiflexible RPs often tend to align *parallel* to one another, forming stacked aggregates. Below, we examine the relationship between the structural characteristics of such stacked aggregates and the cavity radius *R*.

**Figure 5:**
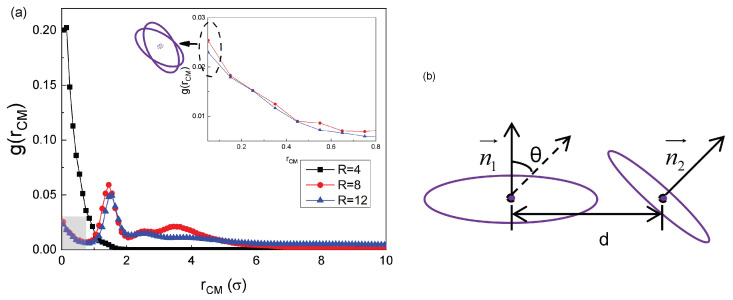
(**a**) Radial distribution function $g(r_{\mathrm{CM}})$ of the CM for semiflexible RPs with $K_{b}=50$ confined within a spherical cavity of varying radius. The inset in panel (**a**) shows a zoom into the gray region of the main plot. (**b**) Schematic illustration of the criterion used to identify and to assign particular RPs to the same stacked aggregate.

We compute the CM radial distribution function $g(r_{\mathrm{CM}})$ for the RPs [23,61,62] with the stiffness $K_{b}=50$ confined within a cavity of varying radius to describe the local CM density characteristics at a distance *r* from the CM of the reference chain; see Figure 5a. This function serves as a key measure of the spatial distribution and of the aggregation state of molecular units.

A nonzero value of $g(r_{\mathrm{CM}})$ at r=0, see the zoomed-in inset of Figure 5a, arises essentially from the coincidence of the CMs of two RPs at a very close spatial neighborhood. This constitutes one of the most prominent topological differences between the RPs and linear chains. In combination with the disc-like quasi-2D structure of semiflexible RPs, it can be inferred that slight interpenetration and quasi-parallel alignment exist between a given RP and its adjacent neighbors; see the schematic inset of Figure 5a for the state at r→0. This arrangement serves as the microscopic prerequisite for the aggregation of RPs and the characteristic of a nonzero g(r) value at r=0 in the stacked structures of semiflexible RPs under unconfined periodic boundary conditions has been widely verified in related studies [23,51,61,62].

For R=8, see the red data points in Figure 5a, the function g(r) exhibits a peak at r≈1.5, which identifies the most probable distance for the other chains to appear around a given semiflexible RP and thus reflects their packing distance. The relatively high height of this peak is indicative of the aggregation degree of the RPs, where a higher peak corresponds to a more ordered arrangement of the RPs at this specific distance. In addition, the peak width represents the range of the ordered segment arrangement: a broad peak implies a large range of short-range order, while a narrow peak indicates a more concentrated order.

In contrast, for R=12, shown by the blue curve and symbols in Figure 5a, the peak is somewhat lower and narrower, with a more concentrated order. This observation is consistent with the simulation snapshots of semiflexible RPs confined by the R=12 sphere, as shown in Figure 2, giving rise to the formation of RP aggregates with multiple orientations.

Under the conditions of strong confinement at, R<5, although the RPs are dispersed inside the cavity, they adhere to the inner wall in a bent conformation, leading to a frequent superposition of their CM positions. This gives rise to a giant peak in the $g(r_{\mathrm{CM}})$ function at the distance $r_{\mathrm{CM}}=0$; see the black curve in Figure 5a. Therefore, the identification of the aggregates should not rely solely on their inter-chain distance, but should also account for the relative orientations of the RPs (e.g., consider the angle between their normal vectors, see Figure 5b).

In Section 3.1, we observed that semiflexible RPs exhibit some disc-like conformations with a well-defined planar orientation and symmetry. This provided a structural basis for a quantitative analysis of the RP aggregates. To define the constituent units of a given aggregate, we introduce now a clear criterion based on the RP conformational features. Specifically, using a definition of aggregation that combines the “spatial proximity and orientational consistency”—which is widely applied in the research on the stacking behavior of semiflexible RPs [23,51,61,62]—we here use the following criterion. (i) Any two semirigid RPs belong to the same stacked aggregate if their CM-CM distance satisfies the condition

(11) $$d_{\text{CM-CM}}<d_{\text{CM-CM}}^{*}=1.5,$$

that is a value corresponding to the peak of the g(r)-profile in Figure 5a discussed above. (ii) The angle between the normal vectors of the RP planes satisfies the condition

(12) $$\theta <\theta^{*}=15^{\circ },$$

see the scheme in Figure 5b.

**Figure 6:**
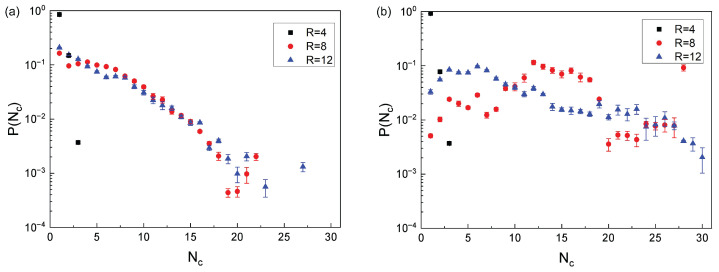
Probability distribution $P(N_{c})$ for an RP to be a part of a stack with $N_{c}$ members, computed for the RPs with the rigidity $K_{b}=10$ (**a**) and $K_{b}=50$ (**b**), confined inside spheres of three different radii.

We compute now the probability distribution, $P(N_{c})$, for an RP to be a part of a stack of $N_{c}$ RPs, namely

(13) $$P\left(N_{c,j}\right)=N_{\mathrm{agg},j}N_{c,j}/N,$$

where $N_{c,j}$ denotes the number of RP chains in the *j*th aggregate, $N_{\mathrm{agg},j}$ represents the number of aggregates containing $N_{c,j}$ RPs. The statistical results show that for $K_{b}=10$, the variations in the cavity radius *R* do not significantly affect the size distribution of the RP aggregates, see Figure 6a. For high bending rigidity ($K_{b}=50$), the RPs possess inherent structural stiffness that resists conformational deformations, making their interchain stacking and aggregation behavior highly sensitive to the size of the spatial spherical confinement. Only when the cavity size matches the particular characteristic dimension required for the ordered structures of the stacked large aggregates of the RPs to be formed does the process take place, while other cavity sizes disrupt this cooperative stacking process because of *incommensurability constrains*. Under a strong confinement, with R=4, the probability of the dispersed RPs is $P(N_{c}=1)\approx 0.91$, consistent with the structural features of the simulation snapshots of the fourth column of Figure 2, computed for the same value of R=4.

In contrast, for much stiffer chains, with $K_{b}=50$, the distribution of sizes of the RP aggregates exhibits a stronger dependence on the cavity radius *R*. Under a strong confinement, with R=4, for instance, the system remains dominated by the dispersed individual non-aggregated chains. However, as the confining radius increases to R=8, the proportion of the dispersed RPs and of small RP aggregates decreases significantly, namely with $P(N_{c}\leq 6)\approx 0.10$.

Meanwhile, the large-size aggregates become the predominant form of compaction, with the probability $P(N_{c}>12)\approx 0.61$, as indicated by the red data points in Figure 6b. At R=8, the cavity size achieves a *critical match* with the geometrical and stiffness characteristics of the $K_{b}=50$ rings: the confined space is sufficient to allow the multiple rings to undergo spatial orientational alignment and to ensure close packing, while the moderate confinement still restricts random chain motion that would disrupt stacking, thus promoting the formation of large-ordered aggregates of the RPs.

From a microstructural perspective, under this condition the average number of the RPs per aggregate reaches a maximum, and the spatial orientational order of all semiflexible RPs also attains its highest level. This leads to the formation of an *optimal* stacked structure characterized by both high chain density and spatial order. A more quantitative analysis of these structures will/can be the subject of our subsequent studies.

As the confining radius *R* increases further, the aggregate distribution undergoes a notable transition. Namely, the proportion of the dispersed RPs begins to rise, reaching $P(N_{c}\leq 6)\approx 0.41$, while both the number and the proportion of large aggregates of the RPs decrease, yielding $P(N_{c}>12)\approx 0.21$, as shown by the data points curve in Figure 6b. This shift can be attributed to a reduced degree of confinement, which allows for greater orientational variability and disrupts large-scale stacking.

A further increase in *R* weakens the spatial constraint on the $K_{b}=50$ rings, leading to the enhanced random thermal motion and to the orientational disorder of the individual chains. The loss of strong confinement-driven alignment eliminates the cooperative RP–RP interactions required to maintain the large stacked aggregates, causing their dissociation into smaller ones and into dispersed individual RPs. This increases the proportion of low-$N_{c}$-species in the system.

**Figure 7:**
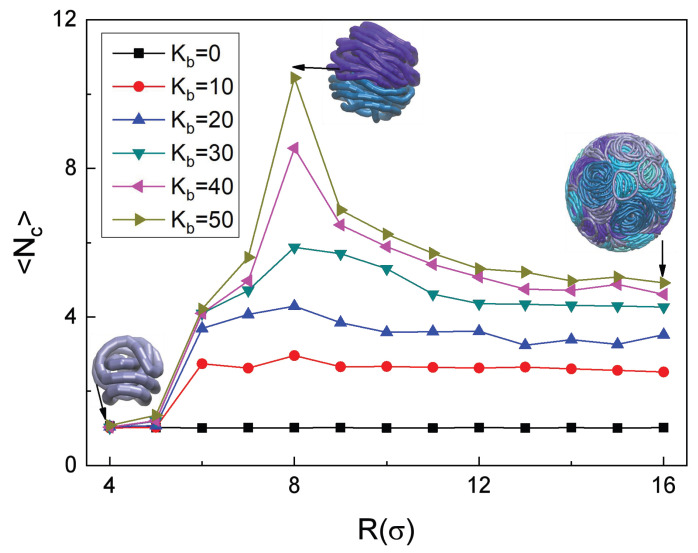
Average number of RPs in stacked aggregates $\langle N_{c}\rangle$ as a function of cavity radius *R*. The color scheme is the same as in Figure 2.

To investigate the effect of confinement degree on the aggregate structures, in Figure 7 we present the results of the quantitative statistical analysis of the *average* number of the RPs per stacked aggregate,

(14) $$\langle N_{c}\rangle =\sum_{j}N_{\mathrm{agg},j}N_{c,j}/\sum_{j}N_{\mathrm{agg},j},$$

computed for different sphere radii *R*.

For the RPs with $K_{b}=10$, the average aggregate size $\langle N_{c}\rangle$ remains largely insensitive to the changes in *R*, see the red curve in Figure 7, consistent with the results of Figure 6a. As the RPs get stiffer, a pronounced dependency $\langle N_{c}\left(R\right)\rangle$ emerges. The increase in $K_{b}$ drastically reduces the conformational adaptability of the RPs, making their stacking behavior highly dependent on the spatial boundary conditions imposed by *R*. Stiffer RPs cannot undergo sufficient local deformations to accommodate confinement changes, so the balance between the conformational stability and the stacking efficiency becomes tightly coupled to the cavity radius *R*. Ultimately, for $K_{b}=50$, the number $\langle N_{c}\rangle$ reaches a maximum of $\langle N_{c}\rangle \approx 10.4$ at R=8. This indicates an optimal balance between the conformations of the RPs and their stacking efficiency under these conditions.

At this critical *R* value, the spatial confinement is moderate enough to restrict the random thermal motion of such rigid RPs and to drive their ordered orientational alignment, while avoiding excessive spatial compression that would induce some conformational distortion of the RPs. This synergy maximizes the cooperative interchain interactions and thus promotes the formation of large stacked aggregates, leading to a peak in the $\langle N_{c}\rangle$ value, as clearly observed in Figure 7.

When R<8, the system becomes strongly confined: the limited internal space thus significantly restricts the conformational freedom of the RPs. To adapt, the RPs undergo local deformations, disrupting the otherwise stable stacked aggregates. This causes a decrease in $\langle N_{c}\rangle$ with a decreasing *R* value.

Under the conditions of extreme confinement, with the sphere radius as small as R=4, the RPs experience severe compression and exhibit considerable distortions, markedly deviating from the ideal disc-like conformation. The inter-chain stacking is dominated by steric hindrance, leading to a predominantly “dispersed state” of individual non-stacked chains. In this scenario, the average aggregate size drops to $\langle N_{c}\rangle \approx 1.07$, approaching thus a single-RP situation.

As the sphere radius, on the contrary, increases to R>12, the boundary effects weaken substantially and the system approaches a regime of a quasi-infinite space. Under these conditions, the stacking of RPs is no longer driven by their spatial constraints, and the aggregates are stable at a small size. The confirmation is presented in Figure 7 both by the data points from the analysis of computer simulations and by visualizing conformations of the RPs in the insets. A larger spatial freedom results in an *anisotropic* distribution of the RP aggregates, with a free-space large-cavity limit amounting to $\langle N_{c}\rangle \approx 5$.

Furthermore, to quantitatively examine the microscopic orientational order of the confined RPs, we use the standard order parameter [100] in d=3 dimensions

(15) $$S=\frac{\langle d\;\cos^{2}\theta -1\rangle }{d-1}=\frac{\langle 3\;\cos^{2}\theta -1\rangle }{2},$$

where θ is the angle between the normal directions of any two RPs. It quantifies the consistency of chain orientations: S=1 indicates the perfect order, while S=0 represents a complete disorder. In our study, semiflexible RPs with L=30 exhibit a spatially parallel orientational order inside a sphere of radius R=8. To accurately compare the global orientational order states of the system, the global order parameter of the RPs is calculated via a statistical approach.

**Figure 8:**
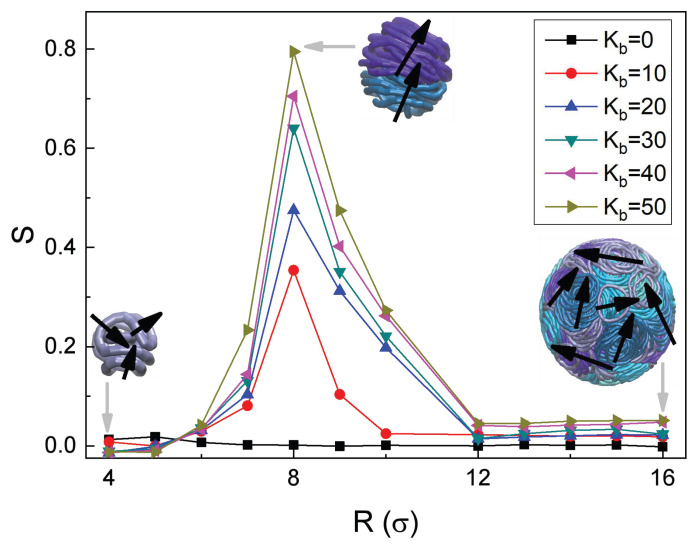
Orientational-order parameter *S* of the RPs versus the sphere radius *R*. The color scheme is the same as in Figure 2.

For the RPs with $K_{b}=50$ and for the cavity radius of R=8, the system of confined RPs exhibits an optimal ordered state. On the one hand, the average number of the RPs per stacked aggregate reaches a maximum under these conditions, reflecting the strongest effective intermolecular aggregation-driving forces. On the other hand, within the formed aggregates, all RPs align parallel to each other along the same direction, with only small orientational deviations. This is illustrated in the middle inset of Figure 8, where the black arrows indicate the directions of the RP planes, resulting in a high order with S≈0.79.

As the confinement radius *R* increases even further—thereby diminishing the degree of spatial constraints imposed onto the enclosed RPs—the entire system naturally shows a significant decrease in its orientational order. The increased free volume within the confining sphere facilitates freedom of the RPs, causing the RP aggregates to lose their regularly aligned distribution and, instead, to exhibit rather *anisotropic* spatial features, see the right inset of Figure 8. At $R>3R_{g}$, the confinement becomes too weak to restrict the random motion of the RPs: the order parameter decreases to S≈0.05, which is indicative of a nearly completely disordered aggregate. The number of the RPs in such aggregates are—despite such a disorder—still given in this large-*R* limit by the values in Figure 7.

In contrast, under strong confinement with R<8, the excessive external spatial constraints induce a significant compression on the RPs and a reduction of their available conformations. This leads to significant distortions from the ideal circular-ring structure, causing the deformations disrupting the regular arrangement within the RP aggregates. The aggregates in this limit are composed, however, of progressively smaller numbers of the RPs, as follows from the data of Figure 7 in this *R*-regime. Consequently, the orientational order is generally lower than that at the optimal-*S* value, which is achieved—at the chosen other parameters, such as the length of the RPs *L*, the number of RPs *N*, and their overall density ρ—at R=8 and continues to decline as *R* decreases further.

For the case of R=4, the order parameter drops to S≈0.01. This result not only reflects a highly dispersed, non-aggregated spatial distribution of the RPs, as seen from Figure 7 at these *R* values, but also indicates completely random inter-chain orientations with no effective cooperative alignment that would stem from the external confinement. This is consistent with a fully disordered state of this mixture of the RPs. Naturally, for other values of *L*, *N*, and ρ, the maximum in the *S* parameter might be reached at *R* values different from R=8 obtained above, but the overall shape of the S(R) curve is still expected to have a similar shape and describe similar qualitative features of the variation of RP orientations with varying degrees of external confinement.

## 4. Conclusions

To get deeper insights into the conformational behavior and the aggregation characteristics of the RPs under external spherical confinement, we employed a coarse-grained model combined with the molecular dynamics computer simulations of a system consisting of the RPs at a relatively high monomer-number density confined within a rigid spherical container. By systematically varying the radius of the external sphere *R*, we quantified how *R* influences the conformational properties of the RPs. The results of the simulations revealed that as the bending energy of the RPs increases, their conformations undergo distinct transitions. Namely, fully flexible RPs adopt compact coil-like conformations driven by maximization of entropy; the RPs with an increased bending energy exhibit elongated oblate conformations; and, lastly, the RPs with a high bending energy drive the system into well-defined disk-like conformations.

Meanwhile, some characteristic structures of the aggregated RPs emerge—the stacked aggregates formed by the adjacent RPs aligning nearly parallel to one another—exhibiting notable coupling with the conformational transitions of the chains. Under a strong confinement at $R<2R_{g}$, the compressive effect of the external cavity induces significant deformation and twisting of the RPs, disrupting the ordered stacked aggregates and markedly reducing the overall structural order. As the container size reaches $R\approx 2R_{g}$, a balance is reached between the confinement strength and the chain mobility. In this regime, the average number of the RPs within stacked aggregates reaches its maximum value, and the spatial orientation of the aggregates shows high uniformity, resulting in a system-wide orientational order parameter reaching a high value of S≈0.79. As the cavity radius increases further, i.e., for $R>2R_{g}$, the semiflexible RPs gain greater mobility that leads to increased randomness in the segmental motion and to diversified orientational distribution of the RP aggregates. Finally, for the systems with $R>3R_{g}$, the confinement effects weaken substantially, and the orientational order parameter drops to S≈0.05, with the RP aggregates becoming thus randomly oriented.

Our study offers key theoretical insights for regulating the highly ordered stacked semiflexible RPs under spherical confinement. As the main novelty and motivation of the current study—as compared to a number of other studies on a similar subject in the field (see Section 1)—we here conducted a comprehensive and quantitative analysis of the orientations of the RPs inside a spherical cavity. The main focus was on quantifying the properties of *RPs aggregates* in different regions of the cavities of variable sizes. The surface-mediated arrangements of the RP stacks triggered by the curvature of the external cavity on the outside of the shell were examined in detail, as compared to the structures of the RPs near the center of the confining cavity.

Taking nanoreactor design as the *first* example, grafting catalytic molecules or nanoparticles onto the semiflexible RPs and confining them in spherical nanoreactors (at $R∼2R_{g}$) induces ordered ring alignment. The latter regularizes active-site arrangement and boosts catalytic efficiency: e.g., the active sites of palladium nanoparticles form uniform arrays, raising the utilization efficiency by over 60% vs. non-oriented systems. This work thus provides important references for the structural design and functional optimization of micro- and nanoscale polymeric materials.

Regarding possible biophysical applications, as the *second* example, in the process of constructing artificial viral-based gene-delivery vectors, the current results can allow us to precisely tune the vector dimensions. This, in turn, will enable the packing of the genetic material—particularly consisting of rather short circular semiflexible DNAs—into highly ordered arrangements, thereby enhancing its structural stability and reducing the risk of leakage. All these factors will ultimately improve the gene-transfection efficiency. Stacked aggregates of larger sizes, more ordered structures, and higher integrity can effectively mitigate structural damage induced by thermal fluctuations and/or external stress, thereby significantly reducing the probability of spontaneous leakage. In natural systems, for example, the DNA molecules encapsulated within the capsids of bacteriophages P22 and λ show a clear concentric layered and tightly packed structure with highly ordered local alignment, which achieves efficient and leakage-free transmission of the genetic material [72,74,75,76].

## Figures and Tables

**Table 1:** The list of main simulation parameters.

Radius of Cavity (in σ)	Polymer Length (in σ)	Number of Chains	Number of Monomers	Density of Monomers (in $\sigma^{-2}$)
R=4	L=30	N=3	N·L=90	ρ≈0.6
R=8	L=30	N=28	N·L=840	ρ≈0.6
R=12	L=30	N=111	N·L=3330	ρ≈0.6
R=16	L=30	N=282	N·L=8460	ρ≈0.6
R=20	L=30	N=574	N·L=17,220	ρ≈0.6

**Table 2:** Eigenvalues of the gyration tensor $\left\{\lambda_{1},\lambda_{2},\lambda_{3}\right\}$, the square of the radius of gyration $\langle R_{g}^{2}\rangle$, the prolateness *p*, and the asphericity *A* for the semiflexible RPs with the stiffness $K_{b}=50$ for varying radii *R* of the confining sphere.

Cavity Radius (in σ)	$\lambda_{1}$ (in σ)	$\lambda_{2}$ (in σ)	$\lambda_{3}$ (in σ)	$\langle R_{g}^{2}\rangle =\lambda_{1}+\lambda_{2}+\lambda_{3}$ (in $\sigma^{2}$)	Prolateness	Aspherisicity
R=4	3.35251	2.52188	1.79102	7.66541	p=0.11039	A=0.03116
R=8	11.30950	8.85199	0.15014	20.31163	p=−0.80604	A=0.25001
R=12	11.69480	8.15019	0.23992	20.08491	p=−0.60500	A=0.25576
R=16	11.59950	8.18019	0.29450	20.07419	p=−0.62317	A=0.25024
R=20	11.59943	8.18055	0.29287	20.07285	p=−0.62337	A=0.25035

## Data Availability

The original contributions presented in this study are included in the article/Supplementary Material. Further inquiries can be directed to the corresponding author.

## Supplementary Materials

The following supporting information can be downloaded at: https://www.mdpi.com/article/10.3390/polym18050602/s1, Figure S1. Typical snapshots of the RPs of varying rigidity $K_{b}$ confined in a spherical cavity of varying radius *R*; Table S1. The list of all simulation parameters used in the main text; Table S2. Eigenvalues of the gyration tensor for *flexible* RPs with $K_{b}=0$ under spherical confinement with varying radius *R*; Table S3. The same as in Table S2, but for *semiflexible* RPs with $K_{b}=10$; Table S4. The same as in Table S3, but for $K_{b}=20$; Table S5. The same as in Table S3, but for $K_{b}=30$; Table S6. The same as in Table S3, but for $K_{b}=40$.

## Notations and Abbreviations

RP, ring polymer; CM, center of mass; FENE, finite extensible nonlinear elastic; LJ, Lennard–Jones; LAMMPS, Large-Scale Atomic/Molecular Massively Parallel Simulator; SI, Supporting Information.
