# Supplementary material for: Structure of Stacked Aggregates of Semiflexible Rings Under Spherical Confinement: A Computational Study
Source: Polymers (Basel). 2026 Feb 28;18(5):602. doi: 10.3390/polym18050602 (PMC12986555; doi:10.3390/polym18050602)
Supplement: Supplementary file 1 [file polymers-18-00602-s001.zip › polymers-4167232-supplementary.pdf]

SUPPORTING INFORMATION

Structure of stacked aggregates of semiflexible rings under spherical confinement: a computational study

Xiaolin Zhou, Yifan Qin, Youfei Xie, and Andrey G. Cherstvy

S1. Simulation Parameters.

Table S1. The list of all simulation parameters used in the main text.

radius of spherical cavity	Length of the polymer chain	number of polymer chains	total number of monomers	Number-density of monomers
R=4	L=30	3	90	ρ≈0.6
R=5		5	150	
R=6		10	300	
R=7		18	540	
R=8		28	840	
R=9		42	1260	
R=10		61	1830	
R=11		83	2490	
R=12		111	3330	
R=13		144	4320	
R=14		184	5520	
R=15		229	6870	
R=16		282	8460	
R=17		343	10290	
R=18		411	12330	
R=19		488	14640	
R=20		574	17220	

S2. Simulation Snapshots of Spherically Confined Rings.

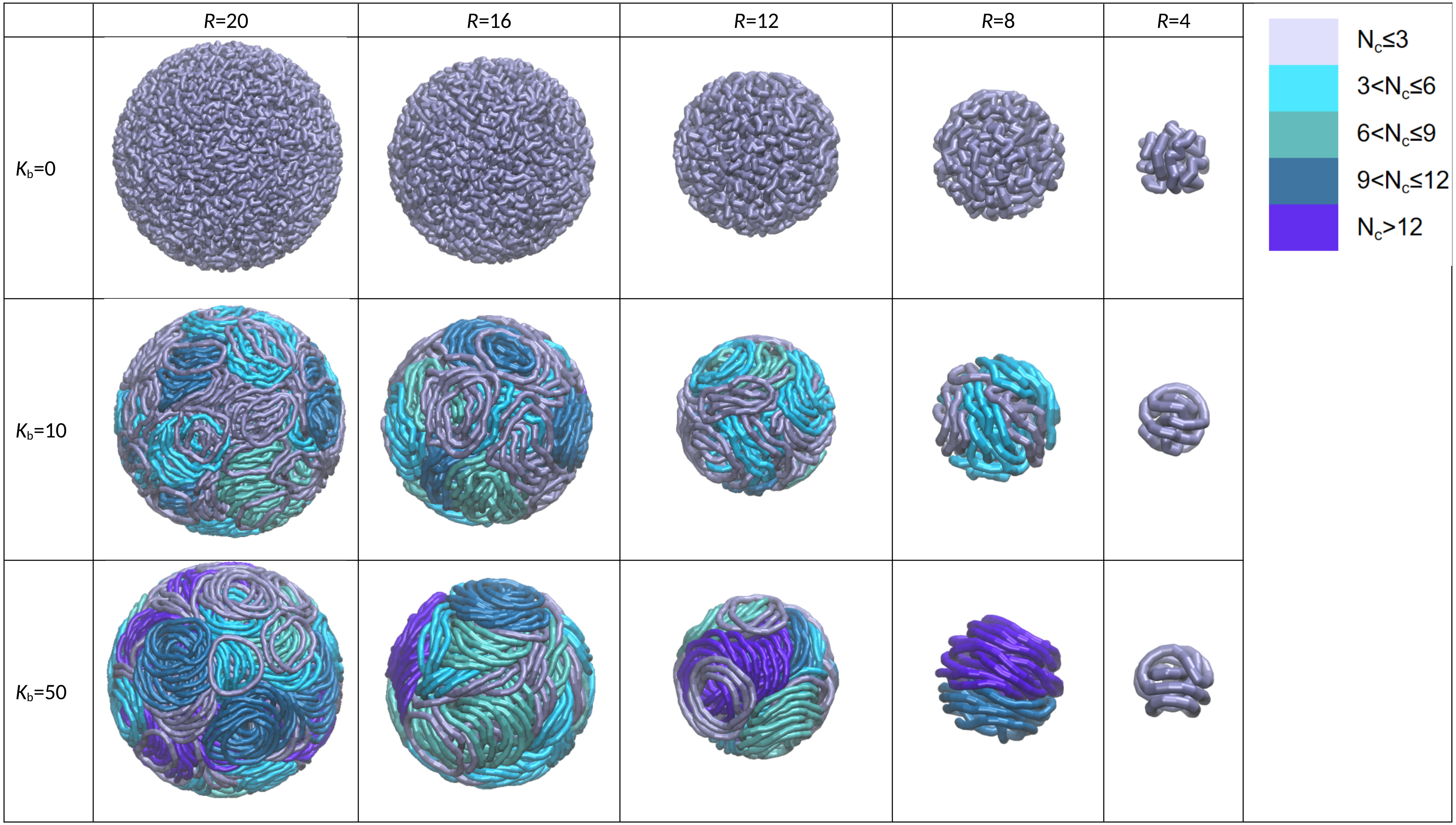

Figure S1. Typical snapshots of the RPs of varying rigidity K_b confined in a spherical cavity of varying radius R.

S3. Eigenvalues of the Gyration Tensor

Table S2. Eigenvalues of the gyration tensor for *flexible* RPs with K_b=0 under spherical confinement with varying radius *R*.

radius	λ_1	λ_2	λ_3	$R_g^2=\lambda_1+\lambda_2+\lambda_3$	R_g	p	A
R=4	2.54124	1.39156	0.59731	4.53011	2.12841	0.31031	0.13964
R=5	2.98009	1.46015	0.51978	4.96002	2.22711	0.39450	0.18795
R=6	3.05360	1.44677	0.50157	5.00194	2.23650	0.43112	0.19961
R=7	3.11196	1.44025	0.50120	5.05341	2.24798	0.46345	0.20544
R=8	3.12174	1.41260	0.47212	5.00646	2.23751	0.47754	0.21596
R=9	3.07960	1.40261	0.49120	4.97341	2.23011	0.48591	0.20907
R=10	3.13171	1.42020	0.50120	5.05311	2.24791	0.49398	0.20940
R=11	3.10196	1.41262	0.50330	5.01788	2.24006	0.49235	0.20719
R=12	3.11746	1.43813	0.52509	5.08068	2.25404	0.48564	0.20095
R=13	3.12314	1.43262	0.52311	5.07887	2.25364	0.49267	0.20247
R=14	3.11243	1.42021	0.50127	5.03391	2.24364	0.48645	0.20770
R=15	3.11196	1.43026	0.50114	5.04336	2.24574	0.47480	0.20656
R=16	3.14175	1.43646	0.52745	5.10566	2.25957	0.49880	0.20272
R=17	3.14218	1.44913	0.52745	5.11875	2.26247	0.48477	0.20138
R=18	3.15017	1.45065	0.52745	5.12826	2.26457	0.48624	0.20190
R=19	3.15331	1.44065	0.52745	5.12140	2.26305	0.49862	0.20326
R=20	3.16369	1.45577	0.52911	5.14857	2.26905	0.48703	0.20214

Table S3. The same as in Table S2, but for *semiflexible* RPs with K_b=10.

radius	λ_1	λ_2	λ_3	$R_g^2=\lambda_1+\lambda_2+\lambda_3$	R_g	p	A
R=4	3.27020	2.19980	1.35203	6.82203	2.61190	0.19938	0.05956
R=5	5.50050	3.31016	1.46948	10.28014	3.20627	0.14955	0.11561
R=6	9.23091	5.11980	0.93837	15.28908	3.91013	-0.01469	0.22064
R=7	10.26059	5.63531	0.50150	16.39740	4.04937	-0.09010	0.26590
R=8	10.44664	6.04749	0.53737	17.03150	4.12692	-0.19270	0.25495
R=9	10.71457	5.58765	0.54979	16.85201	4.10512	0.01517	0.27288
R=10	10.81246	5.75355	0.43256	16.99857	4.12293	-0.04371	0.27971
R=11	10.74812	5.62367	0.40213	16.77392	4.09560	-0.01625	0.28533
R=12	10.74876	5.62156	0.40540	16.77572	4.09582	-0.01490	0.28512
R=13	10.75550	5.60151	0.40499	16.76200	4.09414	-0.00712	0.28598
R=14	10.75550	5.60150	0.40124	16.75824	4.09368	-0.00774	0.28632
R=15	10.79923	5.62210	0.40526	16.82659	4.10202	-0.00662	0.28618
R=16	10.75500	5.59100	0.40621	16.75221	4.09295	-0.00348	0.28622

R=17	10.81430	5.60191	0.41367	16.82988	4.10242	0.00402	0.28643
R=18	10.80137	5.61236	0.40565	16.81937	4.10114	-0.00295	0.28652
R=19	10.79912	5.59100	0.40877	16.79889	4.09864	0.00432	0.28692
R=20	10.80216	5.70124	0.40313	16.90652	4.11175	-0.03284	0.28379

Table S4. The same as in Table S3, but for K_b=20.

radius	λ_1	λ_2	λ_3	$R_g^2=\lambda_1+\lambda_2+\lambda_3$	R_g	p	A
R=4	3.27760	2.24400	1.36000	6.88160	2.62328	0.13462	0.05836
R=5	5.51776	3.36000	1.47360	10.35136	3.21735	0.11590	0.11465
R=6	9.94956	5.52490	0.95996	16.43442	4.05394	-0.02702	0.22442
R=7	11.18856	6.14981	0.49950	17.83787	4.22349	-0.09890	0.26960
R=8	10.88888	7.24472	0.33143	18.46503	4.29710	-0.50616	0.25301
R=9	11.13077	7.00122	0.25900	18.39099	4.28847	-0.40191	0.26714
R=10	11.32500	7.10135	0.20038	18.62673	4.31587	-0.40245	0.27269
R=11	11.25332	7.00464	0.23840	18.49636	4.30074	-0.38352	0.27061
R=12	11.49365	7.00921	0.25382	18.75668	4.33090	-0.34137	0.27298
R=13	11.49313	7.13920	0.25371	18.88604	4.34581	-0.37829	0.27012
R=14	11.61000	7.00110	0.21384	18.82494	4.33877	-0.32380	0.27821
R=15	11.61000	7.15245	0.23840	19.00085	4.35900	-0.36370	0.27281
R=16	11.69381	7.14921	0.24384	19.08686	4.36885	-0.34801	0.27372
R=17	11.69825	7.14712	0.24371	19.08908	4.36911	-0.34669	0.27385
R=18	11.69412	7.14412	0.24319	19.08143	4.36823	-0.34660	0.27389
R=19	11.70330	7.14923	0.24324	19.09577	4.36987	-0.34648	0.27391
R=20	11.74182	7.14967	0.24373	19.13522	4.37438	-0.34007	0.27445

Table S5. The same as in Table S3, but for K_b=30.

radius	λ_1	λ_2	λ_3	$R_g^2=\lambda_1+\lambda_2+\lambda_3$	R_g	p	A
R=4	3.40050	2.37992	1.49960	7.28002	2.69815	0.12738	0.05123
R=5	5.93200	3.77402	1.79801	11.50403	3.39176	0.07615	0.09691
R=6	10.22844	5.78850	0.99602	17.01296	4.12468	-0.06608	0.22098
R=7	11.51189	6.46601	0.61080	18.58870	4.31146	-0.12816	0.25840
R=8	11.39702	7.92034	0.30150	19.61886	4.42932	-0.59468	0.25103
R=9	11.57901	7.94180	0.20147	19.72228	4.44098	-0.57764	0.26042
R=10	11.72011	7.84124	0.18917	19.75052	4.44416	-0.53133	0.26477
R=11	11.72126	7.84412	0.18924	19.75461	4.44462	-0.53184	0.26473
R=12	11.75366	7.78294	0.19911	19.73571	4.44249	-0.51056	0.26546
R=13	11.75341	7.81699	0.19923	19.76963	4.44631	-0.51907	0.26485
R=14	11.75366	7.82404	0.19913	19.77683	4.44712	-0.52078	0.26474

R=15	11.70660	7.84001	0.17911	19.72572	4.44136	-0.53406	0.26538
R=16	11.75317	7.82555	0.19940	19.77812	4.44726	-0.52121	0.26468
R=17	11.75388	7.82512	0.19917	19.77817	4.44727	-0.52101	0.26472
R=18	11.75481	7.82574	0.19928	19.77983	4.44745	-0.52100	0.26471
R=19	11.75392	7.82529	0.19927	19.77848	4.44730	-0.52103	0.26471
R=20	11.75324	7.82511	0.19926	19.77761	4.44720	-0.52110	0.26470

Table S6. The same as in Table S3, but for K_b=40.

radius	λ_1	λ_2	λ_3	$R_g^2=\lambda_1+\lambda_2+\lambda_3$	R_g	p	A
R=4	3.29975	2.44820	1.70135	7.44930	2.72934	0.11316	0.03458
R=5	6.16994	3.99410	1.91330	12.07734	3.47525	0.03866	0.09318
R=6	10.55971	6.01400	1.04295	17.61666	4.19722	-0.07732	0.21902
R=7	11.96436	6.82428	0.64151	19.43015	4.40796	-0.15868	0.25541
R=8	11.23520	8.39600	0.19520	19.82640	4.45268	-0.73122	0.25083
R=9	11.75300	8.24995	0.11711	20.12006	4.48554	-0.62678	0.26408
R=10	11.75414	8.05995	0.21010	20.02419	4.47484	-0.57676	0.26004
R=11	11.75421	8.05900	0.21032	20.02353	4.47477	-0.57651	0.26003
R=12	11.75398	8.05981	0.21033	20.02412	4.47483	-0.57674	0.26002
R=13	11.75221	8.04911	0.21030	20.01162	4.47344	-0.57450	0.26017
R=14	11.75408	8.05926	0.21030	20.02364	4.47478	-0.57659	0.26003
R=15	11.75411	8.06293	0.21028	20.02731	4.47519	-0.57745	0.25998
R=16	11.75499	8.05928	0.21029	20.02456	4.47488	-0.57645	0.26004
R=17	11.75414	8.06122	0.20028	20.01564	4.47388	-0.57780	0.26075
R=18	11.75456	8.05917	0.21031	20.02404	4.47482	-0.57649	0.26004
R=19	11.75478	8.06218	0.21032	20.02728	4.47518	-0.57717	0.25999
R=20	11.75424	8.05944	0.21032	20.02400	4.47482	-0.57661	0.26003
